# Measuring minds across generations: a theoretical framework on psychometric validity, interpretive frameworks, and contextual norming in cross-cohort psychological research

**DOI:** 10.3389/fpsyg.2026.1794790

**Published:** 2026-07-09

**Authors:** Ismail Dergaa, Nasr Chalghaf, Wissem Dhahbi, Khadijeh Irandoust, Halil İbrahim Ceylan, Valentina Stefanica, Imed Chokri, Raul Ioan Muntean, Chamseddine Barki, Hanene Boussi Rahmouni

**Affiliations:** 1 High Institute of Sport and Physical Education of Ksar Said, University of Manouba, Manouba, Tunisia; 2 Physical Activity, Sport, and Health, UR18JS01, National Observatory of Sport, Tunis, Tunisia; 3 High Institute of Sport and Physical Education of Gafsa, University of Gafsa, Gafsa, Tunisia; 4 High Institute of Sport and Physical Education of El Kef, University of Jendouba, El Kef, Tunisia; 5 Training Department, Qatar Police Academy, Police College, Doha, Qatar; 6 Department of Sport Sciences, Imam Khomeini International University, Qazvin, Iran; 7 Physical Education and Sports Teaching Department, Faculty of Sports Sciences, Ataturk University, Erzurum, Türkiye; 8 National University of Science and Technology Politehnica Bucharest, University Center Pitesti, Pitesti, Romania; 9 Department of Physical Education and Sport, Faculty of Law and Social Sciences, University “1 Decembrie 1918”, Alba Iulia, Romania; 10 Research Laboratory of Biophysics and Medical Technologies, Higher Institute of Medical Technologies of Tunis, University of Tunis El Manar, Tunis, Tunisia

**Keywords:** artificial intelligence, clinical interpretation, contextual norming, cross-generational research, differential item functioning, generation Alpha, generation Z, interpretive frameworks

## Abstract

**Background:**

Psychological research increasingly compares mental health outcomes across generations, with studies reporting elevated anxiety and depression in Generation Z and Alpha. Current instruments, interpretive frameworks, and normative standards were developed using language and contextual assumptions from earlier cohorts developing in distinct technological, economic, and social ecosystems.

**Aim:**

This Hypothesis and Theory article proposes to (i) evaluate measurement validity and interpretive framework issues in cross-generational research, (ii) analyze how contexts shape construct meaning across cohorts, (iii) examine artificial intelligence’s impact on Generation Alpha assessment, and (iv) develop methodological frameworks for generation-specific assessment.

**Methods:**

This manuscript presents a Hypothesis and Theory contribution. We conducted a broad theoretical analysis drawing on literature identified through PubMed, PsycINFO, and Web of Science using the terms “generational differences,” “measurement invariance,” “cohort effects,” “differential item functioning,” “psychological assessment,” and related terms. Articles were selected based on theoretical relevance to construct validity, psychometric principles, and generational context. Selection followed expert judgment rather than a formal systematic protocol, consistent with the Hypothesis and Theory format.

**Results:**

Current instruments appear to suggest systematic linguistic drift, with identical items potentially measuring different constructs across generations. Interpretive frameworks fail to account for context-dependent meanings, leading to identical scores representing different psychological realities. Economic transformations reshaped how achievement is defined and assessed across cohorts, with each generation calibrating success relative to the material and social conditions of its formative years. Technology integration from analog to AI-integrated cognition may require adapted assessment frameworks. Generation-specific pathological presentations may be emerging, with corresponding diagnostic categories absent. Differential Item Functioning analyses reveal items operate differently across cohorts, yet uniform interpretations persist. Clinical cut-offs may misclassify generations when responses reflect adaptive functioning.

**Conclusion:**

Cross-generational comparisons using standardized instruments with uniform frameworks may lack fundamental validity. These findings support calls for generation-appropriate assessment, context-sensitive interpretation, cohort-specific norms, and mandatory testing of measurement invariance before cross-generational comparisons.

## Graphical abstract

**Figure:**
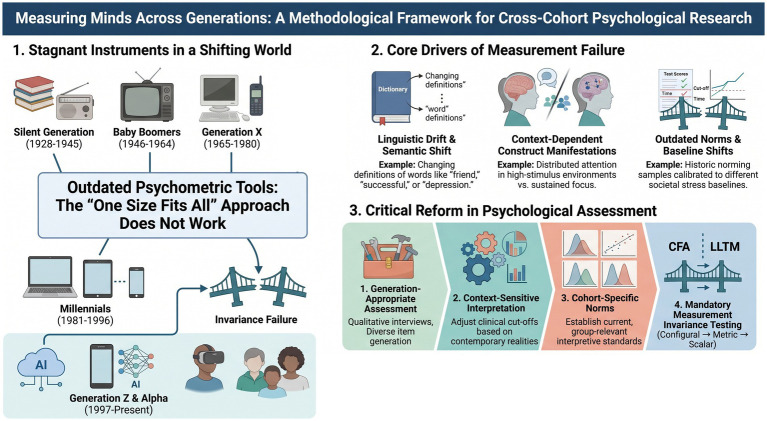
Conceptual framework of cross-generational psychometric assessment, illustrating stagnant instruments applied across generational cohorts from the Silent Generation to Generation Z/Alpha, core drivers of measurement failure (Linguistic Drift and Semantic Shift, Context-Dependent Construct Manifestations, and Outdated Norms and Baseline Shifts), and proposed methodological reforms including Generation-Appropriate Assessment, Context-Sensitive Interpretation, Cohort-Specific Norms, and Mandatory Measurement Invariance Testing.

## 1 Introduction

Psychological research increasingly focuses on generational differences, with literature reporting concerning mental health trends across birth cohorts. Studies suggest Generation Z experiences higher anxiety and depression rates than previous generations, while Generation Alpha raises questions about cognitive development in AI-saturated environments (Twenge et al., 2019a,b; Auxier and Anderson, 2021). These findings have sparked public health concerns and clinical interventions targeting younger cohorts. However, a fundamental methodological question remains largely unexamined: do our measurement instruments, interpretive frameworks, and normative standards possess validity when applied across generations developing in radically different contexts?

Psychological assessment instruments embed the linguistic conventions, social norms, and contextual realities of the period in which they were created (Cronbach and Meehl, 1955). The Beck Depression Inventory emerged in 1961, the State–Trait Anxiety Inventory in 1970, and the UCLA Loneliness Scale in 1978 (Beck et al., 1961). These tools captured constructs as experienced in analog, pre-digital societies with stable employment and face-to-face social networks. Subsequent generations encountered smartphones, social media, gig economies, climate awareness, and artificial intelligence during formative periods. The assumption that identical items measure equivalent states across these divergent contexts requires rigorous scrutiny.

Measurement invariance represents a prerequisite for valid group comparisons (Meredith, 1993). When invariance fails, identical scores reflect different underlying constructs, rendering comparisons meaningless. Despite this requirement, cross-generational research rarely tests measurement invariance across birth cohorts (Millsap, 2011). Researchers compare raw scores between generations separated by decades of transformation, assuming construct stability without empirical verification. This practice violates psychometric principles and may generate spurious conclusions about generational trends (Putnick and Bornstein, 2016).

Each generation’s formative years occurred within distinct contexts. The Silent Generation (1928–1945) developed during economic depression and war, Baby Boomers (1946–1964) during prosperity and Cold War, Generation X (1965–1980) during recessions and family changes, Millennials (1981–1996) during internet emergence and financial crises, Generation Z (1997–2012) during smartphone ubiquity and social media saturation, and Generation Alpha (2013-present) during AI integration and pandemic disruption (Strauss and Howe, 1991). These contextual differences fundamentally shape how constructs manifest, how individuals interpret items, what responses indicate about functioning, and what constitutes normative patterns. It should be noted that the generational descriptions in this article are primarily grounded in North American and Western European contexts; readers from other cultural settings should interpret these characterizations with appropriate caution, and the proposed frameworks may require further adaptation for different sociocultural environments.

Linguistic analysis reveals that psychological terminology evolves across generations (Lakoff, 1987). A “friend” was defined by physical proximity for the Silent Generation. In contrast, for Generation Z, it denotes individuals with whom daily digital communication occurs, even if they have never met in person. “Depression” functioned as a serious clinical descriptor for Boomers, but appears in casual Generation Z discourse describing mild sadness. “Anxiety” referenced specific fears for earlier cohorts but includes diffuse existential dread for younger generations (Barlow, 2002). These semantic shifts may compromise the validity of direct-response comparisons based on instruments with fixed terminology.

Based on identified gaps in measurement validity, interpretive framework adequacy, and normative standard applicability across cohorts, this Hypothesis and Theory article proposes to (i) systematically evaluate how technological, economic, and social context evolution affects psychological construct measurement and interpretation, (ii) analyze mechanisms through which linguistic drift, baseline calibration shifts, and developmental changes compromise cross-generational comparison validity, (iii) examine Generation Alpha as a paradigm case where AI integration renders measurement inadequacy particularly salient, and (iv) develop comprehensive methodological frameworks for generation-specific assessment, context-sensitive interpretation, and continuous norming that acknowledges psychological phenomena as historically situated. We propose a necessary distinction between universal core psychological capacities and their historically situated manifestations. While fundamental human capacities remain stable, environmental triggers and behavioral operationalizations vary profoundly across cohorts. We advance the central hypothesis that cross-generational measurement validity is systematically undermined by linguistic drift, context-dependent construct change, and normative obsolescence. This hypothesis predicts that mandatory measurement invariance testing will reveal significant violations across major psychological instruments, and that generation-specific assessment development will produce superior construct validity indices relative to the standard practice of applying older instruments without cohort-level recalibration.

Literature selection: This manuscript draws on literature identified through PubMed, PsycINFO, and Web of Science using the terms “generational differences,” “measurement invariance,” “cohort effects,” “differential item functioning,” “psychological assessment,” and related terms. Articles were selected based on their theoretical relevance to the central propositions advanced: that cross-generational measurement validity is systematically compromised by linguistic drift, contextual change, normative obsolescence, and DIF. Priority was given to empirical studies testing measurement equivalence across cohorts, seminal psychometric texts, and influential generational research. No date restrictions were applied. As a Hypothesis and Theory contribution, literature selection reflects expert judgment aligned with the theoretical framework of this manuscript, rather than a formal systematic search protocol. The conceptual framework proposed in this article is summarized in Graphical abstract.

## 2 Generational contexts and psychological development

### 2.1 The silent generation (1928–1945): foundation in scarcity and conflict

The Silent Generation developed during the Great Depression and World War II, experiencing childhood marked by economic scarcity, collective trauma, and existential threats to survival (Elder, 1974). Food rationing, housing instability, and family separation characterized their formative years. Communication technology consisted of radio and newspapers, limiting exposure to information to curated broadcasts and daily print media. Social organization centered on tight-knit physical communities, religious institutions, and clear hierarchical structures (Putnam, 2000).

These contextual factors are theorized to have shaped distinctive psychological patterns, including stoicism, emotional restraint, deferred gratification, and institutional trust. Mental health vocabulary remained limited, with psychological distress often somatized or attributed to moral weakness rather than medical conditions (Ryder, 1965). The concept of “anxiety” referenced concrete survival threats, while “depression” indicated severe melancholia requiring institutionalization. Social relationships developed exclusively through physical proximity, making “loneliness” synonymous with geographical isolation.

Current psychological instruments encounter interpretive challenges when applied to this cohort. Questionnaire items asking about “feeling hopeless about the future” must be calibrated against a cohort that experienced actual civilizational collapse (Russell et al., 1978). Their baseline for anxiety involves genuine existential threats rather than diffuse worry characterizing modern anxiety disorders. Self-report measures of social support assume church attendance, neighborhood integration, and multi-generational household structures that no longer predominate.

### 2.2 Baby boomers (1946–1964): prosperity and cultural transformation

Baby Boomers were born during an unprecedented economic expansion, during which median household income in the United States doubled between 1950 and 1970 (Piketty and Saez, 2003). The GI Bill enabled mass college attendance, transforming higher education from elite privilege to middle-class expectation. Suburbs proliferated, offering affordable homeownership to young families. Television emerged as a dominant medium, creating shared cultural experiences (Bogart, 1972).

This generation exhibited idealism, optimism about social progress, and belief in meritocracy. Work became central to identity formation, with career advancement defining success (Easterlin, 1987). Divorce rates increased dramatically, thereby normalizing diversity in family structures. This generation pioneered widespread psychotherapy utilization, reducing mental health stigma. The term “midlife crisis” emerged to describe their specific developmental challenges.

Psychological assessment of Boomers must account for their unique relationship with achievement and success. Questionnaire items about career satisfaction assume accessible advancement ladders and pension security (Hirsch, 2004). Homeownership questions presume market accessibility that requires modest down payments—social comparison operates within local communities, contrasting sharply with global comparisons enabled by later internet connectivity. Depression and anxiety manifested within contexts of material security but social upheaval.

### 2.3 Generation X (1965–1980): skepticism and self-reliance

Generation X emerged during economic recessions, corporate downsizing, and instability in family structures. Many became “latchkey children” with both parents working, experiencing unprecedented childhood independence (Coupland, 1991). The AIDS epidemic, the Challenger disaster, and the Watergate scandal promoted institutional skepticism. MTV revolutionized media consumption through rapid-cut visual content; personal computers and video games introduced early digital technologies (Prensky, 2001).

This cohort is characterized in the generational literature as having developed pragmatism, ironic detachment, and self-reliance as core psychological traits. Trust in institutions, corporations, and government declined markedly (Howe and Strauss, 1993). Work-life balance has become increasingly important as corporate loyalty has proven unrewarding. Mental health awareness increased, though cynicism toward therapeutic culture persisted.

Psychological assessment encounters calibration issues with Generation X around trust, authority, and institutional relationships. Loneliness scales that assume community integration may miss this cohort’s comfort with independence and smaller social circles (McPherson et al., 2006). Achievement motivation measures designed for Boomers’ optimistic meritocracy fail to capture Generation X’s strategic pragmatism. Their “normal” baseline is theorized to include greater skepticism and lower institutional trust than those of previous generations.

### 2.4 Millennials (1981–1996): digital pioneers and economic disappointment

Millennials experienced the emergence of the internet during adolescence and young adulthood, creating a cohort that straddles analog childhoods and digital adult lives. The September 11 attacks shaped their early worldview, followed by prolonged wars (Pew Research Center, 2010). The 2008 financial crisis devastated their career entry, creating long-term economic scarring. Social media platforms emerged, fundamentally altering patterns of social interaction. Student debt escalated dramatically, with average debt loads tripling between 1990 and 2010 (Brown and Caldwell, 2013).

This generation exhibits delayed traditional milestones, including marriage, childbearing, and homeownership. Mental health destigmatization accelerated, with therapy and medication becoming normalized (Arnett, 2000). Anxiety about climate change, economic precarity, and social justice gained prominence. FOMO (Fear of Missing Out) emerged as social media enabled constant awareness of others’ curated experiences.

Measurement challenges multiply with Millennials due to their hybrid analog-digital development. Depression scales asking about social withdrawal fail to distinguish between physical isolation and digital connectivity (Primack et al., 2017). Items on “keeping up with responsibilities” must account for gig-economy work patterns rather than those of stable employment. Questions about future hopefulness are met with rational economic pessimism rather than clinical depression (Case and Deaton, 2020). Achievement measures assuming homeownership accessibility misinterpret systemic barriers as personal failure.

### 2.5 Generation Z (1997–2012): digital natives and constant connectivity

Generation Z constitutes the first cohort with no memory of a pre-smartphone era, having experienced constant connectivity from childhood. Social media platforms dominated their social development, with Instagram, Snapchat, and TikTok shaping identity formation (Boyd, 2014). School shootings and active shooter drills have normalized childhood violence exposure. The COVID-19 pandemic disrupted critical developmental periods, forcing social isolation and remote learning during adolescence (Racine et al., 2021). Climate crisis awareness pervaded their worldview from an early age.

This generation reports the highest levels of anxiety and depression in measured history, though whether this reflects an actual increase, measurement artifacts, or reporting willingness remains contested (Vuorre et al., 2021). Mental health discussion occurs openly, with reduced stigma but potential over-pathologization of normal distress. Social relationships develop through digital platforms, with physical and online friendships carrying equivalent emotional weight (Reich et al., 2012). Identity is theorized to become performative across multiple online personas, potentially complicating the development of an authentic self-concept.

Psychological assessment faces significant validity challenges with Generation Z. Self-esteem measures that assume validation through achievements or relationships fail to account for social media metrics such as followers, likes, and viral content (Vogel et al., 2014). Loneliness scales miss the paradox of feeling isolated despite hundreds of online connections. Depression items about “loss of interest” must distinguish between anhedonia and content overload (Nesi and Prinstein, 2015). Anxiety questionnaires designed for specific phobias cannot capture the diffuse dread of climate catastrophe or economic collapse. Body image measures developed before the rise of filters and influencer culture overlook new dimensions of appearance anxiety (Perloff, 2014).

### 2.6 Generation Alpha (2013-present): AI integration and paradigm shift

Generation Alpha represents a qualitative departure from previous generational transitions, developing with artificial intelligence integrated into cognition from early childhood. Voice assistants like Alexa and Siri may have served as early language interaction models for this cohort (Turkle, 2011). Algorithmic content curation shaped their media consumption before literacy development. The COVID-19 pandemic occurred during critical early development periods, affecting attachment, socialization, and educational foundations. Educational technology, including AI tutors, became standard rather than supplementary (Livingstone and Blum-Ross, 2020).

This generation’s development may increasingly include AI as a cognitive partner rather than a purely external tool. Learning, problem-solving, creativity, and social interaction might increasingly occur with AI assistance from the beginning (Palfrey and Gasser, 2008). Identity formation may occur, in part, through algorithmic feedback that shapes self-concept. Social skills develop through hybrid human-AI environments. Memory and knowledge acquisition strategies differ fundamentally when information access remains constant (Sparrow et al., 2011).

Measurement inadequacy may be especially pronounced among Generation Alpha. Intelligence tests assuming unaided cognition miss the reality of AI-augmented problem-solving (Wechsler, 2008). Memory assessments become meaningless when external information storage is ubiquitous. Creativity measures cannot distinguish between human- and AI-generated content (Amabile, 1996). Social competence evaluations designed for face-to-face interaction fail to capture digital-native social strategies. Existing attachment assessments currently lack frameworks for evaluating AI relationships (Bowlby, 1969).

Generation Alpha presents particularly acute measurement challenges that may also reflect problems existing across all previous generational comparisons. We theorize that the qualitative leap from external tools to integrated AI partners suggests that psychological constructs may lack strict cross-cohort stability, that measurement validity is frequently compromised, and that interpretation likely requires contextual calibration that researchers have often omitted (Clark and Chalmers, 1998).

## 3 Core methodological problems in cross-generational research

### 3.1 Linguistic drift and semantic shift

Psychological assessment relies fundamentally on language, assuming shared semantic understanding between instrument developers and respondents. However, linguistic analysis demonstrates that word meanings evolve across cohorts even when spelling and grammar remain constant (Lakoff, 1987). This semantic drift creates systematic measurement invalidity in cross-generational research.

Table 1 documents specific examples of semantic drift in common psychological assessment terminology based on linguistic analysis and generational discourse studies.

**Table 1:** Semantic drift in psychological assessment terminology across generations.

Term	Silent generation meaning (1928–1945)	Generation Z meaning (1997–2012)	Assessment implication
Friend (Perlman and Peplau, 1981; Parks and Floyd, 1996)	A physically proximate person with regular face-to-face contact and shared local activities.	Online connection across geographic distance, may never meet physically, and daily digital communication.	Loneliness scales misclassify digital connectivity as social isolation; identical scores indicate different levels of social integration.
Depressed (American Psychiatric Association, 1952; Hidaka, 2012)	Severe melancholia requires medical intervention or institutionalization	Mild sadness, temporary disappointment, casual mood descriptor used colloquially	Depression inventories overdiagnose based on lowered threshold for endorsement; semantic drift inflates prevalence estimates
Anxious (Barlow, 2002; Horwitz and Wakefield, 2007)	Specific identifiable fear or worry about a concrete, immediate threat	Diffuse existential dread, generalized unease about the future, chronic background state	Anxiety scales miss new manifestations while detecting old ones; construct validity is compromised across cohorts
Successful (Newman, 1993; Inglehart, 1977)	Stable employment, homeownership, marriage, and children achieved by age 30	Work-life balance, personal fulfillment, social media influence, purpose over profit	Achievement measures assess fundamentally different underlying constructs; economic context confounds motivation assessment
Social (Granovetter, 1973; Baym, 2011)	Face-to-face gatherings, physical proximity, shared in-person activities	Online interaction, digital engagement, curated self-presentation across platforms	Social functioning scales miss the primary social environment; they fail to capture hybrid digital-physical relationships

Consider the term “friend” as it appears in loneliness and social support scales. For the Silent Generation, friendship required physical proximity and regular face-to-face interaction (Perlman and Peplau, 1981). The UCLA Loneliness Scale item “I lack companionship” assumed that relationships were physically present. Generation Z includes friendships formed through online forums, gaming, or shared interests, in which geographic distance and a history of face-to-face meetings are irrelevant (Parks and Floyd, 1996).

When a Generation Z individual endorses “I have few friends” on a loneliness scale, interpretation requires understanding their conceptualization of friendship. They may report few friends while maintaining constant digital contact with a global network, or report many friends, referring to social media followers with whom there is minimal genuine connection (Turkle, 2015). The same item response means fundamentally different things about social integration across cohorts.

Depression terminology underwent a similar evolution. In the 1960s, when the Beck Depression Inventory emerged, “depression” functioned as serious clinical language indicating severe melancholia (American Psychiatric Association, 1952). Generation Z uses “depressed” colloquially to describe mild temporary sadness, with phrases like “I’m so depressed about this exam” indicating frustration rather than clinical symptomatology (Hidaka, 2012).

When questionnaires ask “Do you feel depressed?” the threshold for affirmative responses differs dramatically across generations. Silent Generation members might endorse only severe melancholia, whereas Generation Z individuals might report transient sadness (Cacioppo et al., 2010). Identical endorsement rates across cohorts could reflect decreasing severity or increasing openness. Without accounting for semantic shift, interpretation becomes impossible.

The term “successful” illustrates how economic context shapes the meaning of a construct. For Baby Boomers, success included homeownership, stable employment, marriage, and children, all accessible through moderate effort in expanding economies (Newman, 1993). Millennials redefined success to emphasize work-life balance, experiences over possessions, and purpose over profit, partly in response to economic realities, thereby making traditional markers unattainable (Inglehart, 1977). Generation Z increasingly views success as building personal brands or achieving social media influence. Achievement motivation scales asking about “wanting to be successful” measure different underlying constructs across these cohorts (Deci and Ryan, 2000).

These linguistic shifts violate measurement invariance at fundamental levels. Differential Item Functioning analyses would likely reveal that items function differently across cohorts; such findings may signal possible threats to construct comparability, though further qualitative and validity evidence would be required to determine whether item meaning has genuinely shifted (Chen, 2007). Yet cross-generational research proceeds as though language remains stable, comparing raw scores without accounting for semantic evolution.

### 3.2 Context-dependent construct meanings

Beyond linguistic drift, psychological constructs themselves manifest differently across generations, rendering supposedly universal phenomena generation-specific. Social isolation provides a clear example. Loneliness scales developed in the 1970s and 1980s measured the absence of physical community integration, assuming that social connection required geographic proximity and face-to-face interaction (Wellman et al., 2003).

For the Silent Generation and Baby Boomers, social isolation genuinely meant physical separation from the community. Living alone, lacking local friends, and having infrequent in-person contact indicated a problematic level of disconnection (Rainie and Wellman, 2012). However, Generation Z experiences an entirely different social ecology. An individual might live alone, rarely see people in person, yet maintain constant communication with geographically dispersed friends through text messaging, video calls, online gaming, and social media (Chou and Edge, 2012).

Conversely, Generation Z individuals may have numerous social media connections, regular in-person contact, and busy social calendars, yet report intense loneliness. The performative nature of social media creates superficial interactions masquerading as genuine connections (Harter, 2012). Curated online presentations promote comparison and inadequacy rather than belonging. This “loneliness in crowds” phenomenon has no equivalent in earlier generations’ experiences, yet loneliness scales lack items capturing it.

Attention and concentration similarly depend on context. Attention-deficit/hyperactivity disorder (ADHD) assessments and attention measures assume environments with minimal distractions and tasks requiring sustained focus on a single activity (Barkley, 1997). These assumptions reflected analog environments in which reading books, attending lectures, or completing paperwork were typical demands. Scoring systems classify the inability to sustain single-task focus for extended periods as pathological.

Generation Z developed in high-stimulation environments with constant notifications, multiple simultaneous information streams, and rapid content switching. Their cognitive strategies were adapted to these demands, developing skills in rapid task switching, parallel information processing, and filtering high-volume input (Ophir et al., 2009). Traditional attention measures interpret these adaptations as deficits, potentially misclassifying an entire generation’s cognitive style as disordered. An alternative interpretation suggests that Generation Z exhibits attentional patterns suited to modern environments: reduced sustained single-task focus but enhanced distributed attention and pattern recognition across multiple inputs (Carrier et al., 2015).

Achievement motivation constructs demonstrate similar context-dependence. Scales developed during periods of economic expansion and social mobility measured drive for career advancement, financial success, and status attainment (McClelland, 1961). These goals were realistic for middle-class individuals with moderate effort. High achievement motivation indicated healthy striving, while low scores suggested problematic apathy.

For Millennials and Generation Z facing student debt, housing unaffordability, gig-economy instability, and the climate crisis, traditional achievement markers appear increasingly unattainable. A lack of motivation toward homeownership or career advancement may reflect a realistic assessment of systemic barriers rather than personal deficiency (Case and Deaton, 2020). Conversely, alternative forms of achievement, such as social media influence, creative expression, or activism, may reflect high motivation that traditional scales fail to capture (Côté and Levine, 2002).

Depression constructs reveal profound context-dependence around hopefulness and future orientation. Beck Depression Inventory items include “I feel discouraged about the future” and “I feel that the future is hopeless” (Beck et al., 1996). For Baby Boomers developing in expanding economies with improving living standards, future pessimism indicated depressive cognitive distortion. Their baseline expectation included upward mobility and retirement security.

Generation Z confronts climate models predicting catastrophic environmental change, economic projections suggesting declining living standards, and automation threatening employment stability (Wallace-Wells, 2019). Their “hopelessness” about the future might represent an accurate assessment of documented systemic problems rather than cognitive distortion. Distinguishing clinical depression from rational despair becomes impossible without accounting for contextual realities (Ojala, 2012).

Self-esteem and self-worth constructs similarly depend on sources of validation that vary across generations. The Rosenberg Self-Esteem Scale assesses feelings of “good enough” and “of worth,” assuming internal standards and social feedback from proximal relationships (Rosenberg, 1965). For pre-digital generations, self-esteem derived from achievement, relationship quality, and community regard within limited comparison contexts.

Social media metrics, including followers, likes, comments, and engagement rates, influence Generation Z’s self-esteem. External validation becomes quantified and constant (Chou and Edge, 2012). A global comparison with influencers, celebrities, and curated presentations replaces a local comparison with peers. Self-worth fluctuates with algorithmic visibility and viral potential. Traditional self-esteem scales overlook these dynamics, measuring outdated sources of validation while ignoring dominant contemporary influences on self-concept (Harter, 2012).

These context-dependent meanings challenge the assumption of construct stability required for valid cross-generational comparison. Researchers might measure different phenomena while assuming they assess identical constructs across cohorts.

### 3.3 Baseline calibration and normative standards

Psychological assessment interprets scores relative to normative baselines and classifies responses as normal, elevated, or clinically significant based on population standards. These norms typically derive from validation samples tested decades prior, embedding outdated baselines (Anastasi and Urbina, 1997). As generational contexts shift, normative standards become invalid, potentially misclassifying entire cohorts.

Anxiety measures exemplify this problem clearly. The State–Trait Anxiety Inventory and Generalized Anxiety Disorder scale use cutoff scores established with samples from earlier decades (Spielberger et al., 1983). These populations exhibited different baseline anxiety levels, calibrated to their respective environmental contexts. Silent Generation and Boomer samples faced nuclear threats but lacked sustained exposure to information about global catastrophes. Their baseline anxiety is theorized to have reflected specific situational fears rather than diffuse existential dread.

Generation Z is developing amid 24/7 news cycles that broadcast global disasters, social media amplifying threats, and algorithms that prioritize emotionally arousing content. Their baseline anxiety level may be higher simply due to the information environment, representing adaptive vigilance rather than disorder (Holman et al., 2014). When contemporary samples exceed normative cutoffs established decades ago, interpretation requires determining whether scores reflect increased pathology or a baseline shift. Without recalibration, we risk pathologizing an entire generation’s normal response to genuinely threatening environments (American Psychiatric Association, 2013).

Sleep disturbance provides another clear example. Sleep questionnaires ask about sleep latency, nighttime awakenings, and total sleep duration, with norms established in pre-smartphone eras. The Silent Generation and Baby Boomers typically followed natural circadian rhythms, with limited evening light exposure and no bedtime technology use (Carskadon et al., 1993).

Generation Z bedrooms contain smartphones, tablets, laptops, and LED lighting, with constant connectivity to social networks. Blue light exposure suppresses melatonin, delaying sleep onset (Cajochen et al., 2011). Social media offers numerous opportunities for engagement, which may lead to later bedtimes. FOMO motivates checking devices throughout the night. Their “normal” sleep patterns differ from those of earlier generations, not due to individual pathology but rather to ubiquitous technology that alters environmental conditions (Lemola et al., 2015).

The use of technology itself illustrates baseline calibration problems. Screen time questionnaires classify daily use levels as concerning based on norms from low-technology eras. For Baby Boomers, multiple hours of daily screen time indicated excessive television watching (Robinson et al., 2007). Modern reality requires extensive screen time for work, education, communication, entertainment, and daily functioning. Generation Z may spend 8–10 h per day on screens for legitimate activities, including remote learning, job searching, maintaining relationships, and accessing services. Using outdated norms would classify their entire cohort as addicted (Twenge et al., 2019b).

Social relationships underwent a similar baseline transformation. Friendship inventories and social support scales established norms when in-person interaction was the only method of relationship maintenance. Daily or weekly face-to-face contact indicated healthy relationships, while infrequent contact suggested weak ties (Granovetter, 1973).

Digital communication enables relationship maintenance across geographic distance with minimal effort. Generation Z may have close friendships characterized by frequent daily text communication but infrequent in-person meetings due to distance, work schedules, or economic constraints on travel. Traditional norms would classify these as weak relationships despite emotional intimacy and consistent contact (Rainie and Wellman, 2012).

Career achievement norms became similarly outdated. Measures of job satisfaction, career progress, and occupational success established norms during eras of stable employment, pension systems, and predictable advancement. Career satisfaction for Baby Boomers meant steady advancement, rising salaries, and retirement security, all attainable through organizational loyalty (Hall and Mirvis, 1995).

Generation Z enters the gig economy, contract positions, and frequent job changes, necessitated by precarious employment. Traditional career satisfaction measures penalize adaptation to changing economic conditions (Kalleberg, 2011). A lack of job security, frequent employment transitions, and the absence of retirement planning reflect systemic change rather than individual dysfunction.

Body image norms require comprehensive recalibration for social media generations. Body dissatisfaction scales were developed before photo editing, filters, influencer culture, and algorithm-curated beauty content. Earlier norms reflected dissatisfaction with magazine images and television (Grabe et al., 2008).

Generation Z encounters an abundance of filtered and edited images that present unrealistic beauty standards. Their baseline body dissatisfaction may be higher due to constant comparison with digitally altered images rather than to individual pathology (Tiggemann and Slater, 2013). Norms established pre-Instagram miss that environmental conditions fundamentally changed, potentially classifying universal responses to toxic media environments as individual eating disorder risk.

### 3.4 Differential item functioning across birth cohorts

Differential Item Functioning analysis provides a statistical methodology for detecting items that significantly differ in difficulty across compared groups, conditional on the latent construct level. DIF operates at the individual item level and does not directly appraise overall construct validity; when many items exhibit DIF, however, broader questions about construct comparability may legitimately arise. An item exhibits DIF when individuals with equivalent levels of the latent construct exhibit different response patterns across groups (Embretson and Reise, 2000). In cross-generational research, DIF analysis can reveal whether items function equivalently across birth cohorts.

Table 2 presents selected studies illustrating measurement non-invariance and differential item functioning across generational cohorts. These studies were chosen as illustrative examples rather than as a comprehensive review; priority was given to research examining major psychological instruments and cohort or age-group comparisons directly relevant to the DIF framework advanced here. We specifically selected these constructs (such as depression, anxiety, and narcissism) because they are among the most commonly utilized domains in cross-generational trend research and already possess preliminary evidence of high vulnerability to linguistic and contextual drift.

**Table 2:** Empirical evidence of measurement non-invariance across generational cohorts.

Study	Instrument	Cohorts compared	Key finding	Invariance level achieved
Twenge et al. (2010)	Minnesota Multiphasic Personality Inventory (MMPI) scales	1938–2007 birth cohorts	Birth cohort increases are documented across multiple clinical scales; authors acknowledged the possibility of genuine change alongside potential measurement artifacts	Formal invariance testing not conducted; cross-temporal meta-analytic design precludes confirmatory factor analysis (CFA)-based invariance decomposition.
Donnellan et al. (2009)	Narcissistic Personality Inventory	1980s-2000s college students	No evidence of a narcissism epidemic; prior findings are likely measurement artifacts	Partial scalar invariance
Teresi and Fleishman, (2007)	Depression and anxiety scales	Younger vs. older adults	Substantial DIF on items related to somatic symptoms and future outlook	Metric invariance only
Glaesmer et al. (2008)	Life Orientation Test-Revised (LOT-R)	German population across age cohorts	Optimism items functioned differently across age groups	Partial metric invariance
Meade and Lautenschlager, (2004)	Various personality measures	Simulated cohort data	Demonstrated high rates of Type I error when invariance was violated	N/A (methodological study)

The limited research examining DIF across cohorts consistently demonstrates significant violations (Meade and Lautenschlager, 2004). These findings indicate that identical scores across generations may reflect different psychological states, invalidating direct comparisons. However, the vast majority of cross-generational research proceeds without DIF testing, implicitly assuming invariance without empirical verification (Vandenberg and Lance, 2000).

Depression scales demonstrate clear DIF patterns across cohorts. Studies examining the Beck Depression Inventory across age groups found that items referencing social withdrawal, future outlook, and self-criticism showed significant DIF. Older cohorts required higher depression levels to endorse social withdrawal items, while younger cohorts endorsed these items at lower depression levels (Segal et al., 2008). This pattern likely reflects the fact that reduced in-person socializing indicates more severe pathology in cohorts in which physical interaction was the only social option. In contrast, digital-native cohorts maintain connections through technology, making physical withdrawal less indicative of severe depression.

Items about future pessimism showed opposite DIF patterns, with older cohorts more readily endorsing hopelessness at equivalent depression levels. Younger cohorts required higher depression to endorse future hopelessness, possibly because environmental realities make some future pessimism normative rather than indicating cognitive distortion (Abramson et al., 1989). The same item response suggests different levels of depression severity across cohorts, violating measurement invariance.

Anxiety scales exhibit DIF related to technological anxiety sources. Studies of anxiety inventories found generational differences in item functioning for questions about “feeling nervous” and “worrying about things” (Osman et al., 2001). Younger cohorts endorsed these items at lower anxiety levels, potentially reflecting semantic drift where casual usage reduced the threshold for endorsement. Alternatively, baseline anxiety levels may have increased due to information environments.

The statistical implications of DIF are profound. When significant DIF exists, comparing raw scores across groups produces invalid conclusions (Cheung and Rensvold, 2002). Establishing measurement invariance at the configural, metric, and scalar levels before comparing means is a rigorous, widely recommended approach. Configural invariance requires an identical factor structure across groups. Metric invariance requires equivalent factor loadings. Scalar invariance requires equivalent item intercepts, and only with scalar invariance established can mean comparisons validly proceed (Widaman and Reise, 1997). This CFA-based framework is well established, though it is not the exclusive valid method. Item response theory (IRT)-based DIF detection procedures, particularly Mantel–Haenszel approaches within Rasch or one-parameter logistic (1-PL) frameworks, represent equally rigorous alternatives that estimate item parameters independently of normative populations, providing favorable Type I error control and greater sample-independence than CFA-based methods in cross-generational contexts.

An important distinction must be drawn here. Scalar invariance and DIF analyses assess whether items function statistically equivalently across groups, specifically whether item intercepts and item difficulties are comparable across groups. This provides evidence of an instrument’s internal structural consistency. However, scalar invariance does not, by itself, establish construct validity in the broader psychometric sense. Construct validity concerns whether an instrument actually measures the intended psychological phenomenon across groups, including whether the construct carries equivalent conceptual and linguistic meaning for respondents in each group. The central argument of this manuscript is that psychological terminology and construct manifestations shift across generations, implicating construct validity at this deeper level. DIF and scalar invariance testing are components of the evidence base for construct validity, but they cannot, alone, determine whether an instrument measures the same psychological reality across cohorts separated by decades of technological and social transformation.

Most cross-generational research never tests these prerequisites, proceeding directly to mean comparisons and inferring generational differences from score discrepancies (Vandenberg and Lance, 2000). Given the consistent DIF findings across studies testing invariance, much published research on generational mental health trends likely reflects measurement artifacts rather than genuine psychological differences.

### 3.5 Temporal confounding and the impossibility of replication

Cross-generational research suffers from fundamental temporal confounding that precludes accurate replication. When researchers attempt to replicate studies conducted decades earlier, they inadvertently introduce systematic confounds arising from historical period effects, technological change, and social change (Glenn, 2005). What appears to be replication actually compares populations developing in incomparable contexts, attributing differences to generational characteristics rather than to environmental shifts.

Consider a prototypical example: a study conducted in 2000 examining adolescent social anxiety finds that 15% of participants meet clinical criteria. A 2020 replication using identical instruments reports 28% meeting criteria, prompting conclusions about Generation Z experiencing elevated social anxiety compared to Millennials. However, this interpretation assumes the construct “social anxiety” remained stable across two decades (Schaie, 1965).

The 2000 sample developed social skills primarily through face-to-face interaction, with limited digital communication. Social anxiety manifested as fear of in-person encounters, public speaking, or group settings. The 2020 sample navigated hybrid social environments where online presentation, social media engagement, and digital judgment occurred constantly (Brooks et al., 2020). Their anxiety incorporated fear of negative comments, low engagement metrics, unflattering posts, or cancel culture consequences. Traditional social anxiety scales miss these digital-specific concerns.

The difference between 15 and 28% might reflect genuine anxiety increase, changed anxiety manifestation, improved recognition and reporting, semantic drift in item interpretation, or baseline calibration shifts. Disentangling these possibilities requires methodological sophistication absent from most cross-generational research.

## 4 Generation Alpha and the AI paradigm shift

### 4.1 AI as qualitative departure from previous technological transitions

Previous generational transitions involved quantitative increases in information access, communication speed, or connectivity. Television provided visual information faster than newspapers. Personal computers processed data more efficiently than typewriters. The internet connected people across distances. Social media amplified interaction frequency. These represent linear technological progression, improving existing functions without fundamentally altering cognitive processes.

Generation Alpha potentially confronts a qualitative leap: artificial intelligence capable of thinking, creating, learning, and responding in ways previously unique to humans. This transition resembles the difference between calculators (augmenting calculation) and computers (performing novel computation), or between cars (faster transportation) and aircraft (entirely new modes of mobility). AI does not merely augment human cognitive functions; it performs tasks previously monopolized by humans, potentially requiring a reconceptualization of cognition, creativity, and intelligence.

Language acquisition provides a clear example. Previous generations learned language exclusively through human interaction, with parents, caregivers, and educators providing linguistic input. Generation Alpha encounters voice assistants such as Alexa and Siri as their first language models, which respond to questions, narrate stories, and demonstrate conversational patterns. The cognitive and social implications of AI-mediated language development remain unexplored, yet they clearly differ from those of purely human language acquisition.

Learning and problem-solving underwent a similar transformation. Prior generations developed strategies for information seeking, knowledge retention, and problem decomposition because these skills proved necessary. Generation Alpha can outsource these functions to AI assistants that search information, remember facts, and suggest solutions instantly. The cognitive abilities they develop may emphasize question formulation, answer evaluation, and system management rather than information retention and algorithmic problem-solving.

Creativity historically required human imagination, skill development, and iterative practice. Generation Alpha uses AI systems to generate art, music, stories, and designs from text prompts. Their creative development occurs alongside AI-generated professional-quality output without human-level skill. We hypothesize that the relationship between effort, ability, and innovative production has fundamentally changed, with uncertain implications for creative self-efficacy, artistic identity, and motivation to develop human skills when AI alternatives exist.

Social interaction represents another domain of qualitative shift. Previous generations developed social skills exclusively through human relationships, learning emotional recognition, perspective-taking, and conversational norms from social feedback. Generation Alpha interacts with AI chatbots, virtual assistants, and algorithm-driven content from early childhood. These interactions differ fundamentally from human socialization: AI never tires, judges, or rejects; it optimizes engagement rather than authentic connection; it responds predictably rather than with human complexity.

The distinction between an external tool and an integrated cognitive partner crystallizes this qualitative difference. Baby Boomers used calculators as external aids, picking them up for specific tasks, then setting them aside. Generation Alpha’s AI integration resembles internalized cognitive processes: it is constantly available and automatically consulted. This integration could conceptualize human-AI systems where cognitive processes are theoretically distributed across biological and artificial substrates, potentially making the individual assessment of either component miss the functional reality.

### 4.2 New psychological constructs requiring assessment

Generation Alpha’s AI-integrated development creates entirely new psychological phenomena that require conceptualization and measurement. Table 3 lists emerging constructs for which no current assessment frameworks exist.

**Table 3:** Emerging psychological constructs in Generation Alpha requiring assessment development.

Construct	Definition	Assessment need	Clinical relevance
AI relationship quality	Depth, security, and functional impact of bonds with AI companions and virtual assistants	Measure emotional investment, dependency levels, and impact on human relationship formation.	Distinguish healthy AI interaction from problematic over-reliance; guide intervention when AI relationships substitute for human connection.
Cognitive sovereignty	Sense of ownership and intentional control over one’s thinking processes when AI assistance is ubiquitous.	Assess awareness of AI influence, intentionality in AI use, and metacognitive understanding.	Identify individuals with diminished agency and develop interventions that promote deliberate AI collaboration.
Synthetic content literacy	Ability to distinguish AI-generated from human-created content and critically evaluate AI outputs	Test detection skills, understanding of AI limitations, and appropriate skepticism	Screen for vulnerability to AI-generated misinformation; assess reality testing in AI-saturated environments
Digital authenticity comfort	Psychological ease with AI-assisted self-presentation and content creation	Measure anxiety about authenticity, impostor feelings, and identity coherence in relation to AI use.	Address distress from blurred boundaries between human and AI contributions to self-presentation.
Extended intelligence management	Metacognitive skill in strategically using AI augmentation to maximize human-AI system performance	Evaluate AI tool selection, output evaluation, and integration of AI assistance.	Distinguish over-reliance from under-utilization; optimize human-AI collaboration.
AI-mediated self-concept	Identity formation influenced by algorithmic feedback and AI-curated social environments	Assess self-concept stability, awareness of algorithmic influence, and the maintenance of authentic identity.	Address identity fragmentation; intervention when algorithmic optimization distorts self-knowledge.
Existential displacement anxiety	Distress from AI performing tasks previously defined by human uniqueness, worth, and purpose	Measure concerns about AI competition, fears of irrelevance, and existential questioning.	Identify at-risk individuals; develop meaning-making interventions for the AI era.

AI Relationship Quality is proposed here as a critical emerging construct requiring future empirical investigation. Generation Alpha forms relationships with virtual assistants, AI companions, and chatbots that provide emotional engagement, companionship, and support. These relationships differ from traditional friendships (mutual, between equals) and parasocial relationships (one-sided, with media figures). They involve interactive, responsive, seemingly emotionally attuned entities that remember preferences and simulate caring. Assessment requires measuring AI relationship quality, emotional investment, dependency levels, and impacts on human relationship formation.

Existing attachment and relationship measures assume that the targets are human. Items about “turning to someone for comfort” or “having someone who understands” require clarification whether AI companions count. For Generation Alpha, excluding AI relationships from social support assessments may miss emerging sources of support. Including them without a conceptual framework for understanding their psychological significance generates measurement without meaning.

### 4.3 Interpretive framework challenges for generation alpha assessment

Even existing constructs measured by traditional instruments require entirely new interpretive frameworks accounting for AI integration. Table 4 demonstrates interpretive ambiguities when applying standard assessment to AI-integrated functioning.

**Table 4:** Interpretive challenges for traditional constructs in Generation Alpha.

Traditional construct	Standard assessment assumption	Generation alpha reality	Interpretive ambiguity
Intelligence (IQ)	Individual problem-solving without external assistance; scores reflect innate cognitive capacity	AI-augmented problem-solving from early development; “unaided” capacity is an artificial constraint	Does a low score indicate cognitive deficit or poor AI management? Does a high score reflect individual ability or system optimization?
Attention and executive function (ADHD)	Sustained single-task focus; internal attention management	External attention management via AI reminders and task systems; distributed attention across multiple inputs	Is poor unaided attention pathological or adaptive? Should the assessment measure AI-supported or only independent functioning?
Social skills	Face-to-face interaction quality; emotional recognition in physical presence	Hybrid digital-physical social environments; text-based emotional communication	Do limited face-to-face skills but strong digital communication indicate a deficit or adapted competence?
Memory	Internal information storage and retrieval	External information storage via AI; meta-memory for accessing information	Should memory assessment test internal storage when external storage is ubiquitous and adaptive?
Creativity	Original human-generated ideas and artistic production	AI-assisted ideation and content generation from early childhood	Does AI-assisted creativity indicate a lack of originality or sophisticated tool use? How to assess human contribution?

Intelligence assessment exemplifies the interpretive crisis. IQ tests and cognitive measures assume that individuals can solve problems without external assistance. The instructions specify that tasks be completed independently, and scores reflect unaided cognitive capacity. This framework presumes intelligence exists within individuals rather than being distributed across human-tool systems.

We hypothesize that Generation Alpha’s intelligence might develop alongside instantly available AI capable of complex reasoning. Their “unaided” cognitive capacity represents an artificial constraint that has never been encountered in their actual functioning. Measuring intelligence without AI assistance is like assessing the mathematical ability of pre-calculator generations without pencil and paper. The measure captures something, but whether it captures functionally relevant intelligence remains questionable.

## 5 Methodological solutions and future directions

### 5.1 Generation-specific assessment development framework

Addressing cross-generational measurement validity requires the systematic development of generation-specific assessment tools, acknowledging that psychological constructs manifest differently across cohorts. Table 5 outlines the recommended comprehensive development process.

**Table 5:** Framework for generation-specific assessment development and implementation.

Phase	Activities	Methods	Outcome
1. Qualitative foundation	In-depth exploration of construct experiences within target generation	Focus groups, semi-structured interviews, discourse analysis, and social media linguistic analysis	Generation-specific item pool reflecting contemporary language, salient concerns, and novel manifestations
2. Item development	Create parallel forms that include core universal items and generation-specific items.	Expert review, cognitive interviewing, readability assessment, cultural adaptation	Measurement tools capturing both universal constructs and generation-unique expressions
3. Psychometric validation	Establish reliability, factor structure, and convergent/discriminant validity within generation.	Confirmatory factor analysis, reliability coefficients, criterion validation with generation-relevant outcomes	Psychometrically sound instruments validated for a specific cohort
4. IRT linking	Identify anchor items functioning equivalently across cohorts; establish a standard scale.	Differential Item Functioning analysis, item calibration, and test equating procedures.	Ensure score comparability across generations while maintaining generation-specific content.
5. Normative data collection	Gather representative samples from each generation; establish cohort-specific norms.	Stratified sampling, continuous norming, percentile calculation, and cutoff determination	Current, generation-appropriate interpretive standards
6. Ongoing monitoring	Regular re-norming, item analysis, and invariance testing as cohorts age	Longitudinal data collection, periodic DIF analysis, norm updates every 2–3 years	Maintained measurement accuracy, accounting for both developmental and period effects

The development process must begin with comprehensive qualitative research within each generational cohort. Focus groups, interviews, and discourse analysis should explore how generations experience and describe psychological phenomena. This research identifies generation-specific language, salient concerns, and novel manifestations of traditional constructs. For Generation Z, qualitative work would reveal social media-specific anxieties, digital identity concerns, and climate-related existential dread absent from earlier cohorts. Qualitative research on Generation Alpha should explore AI relationship experiences, concerns about authenticity, and cognitive strategies in AI-augmented contexts.

Parallel form development produces generation-specific instruments that measure equivalent constructs with cohort-appropriate content. This approach maintains construct consistency while adapting measurement to generational contexts. A depression scale would include core neurovegetative symptoms that are consistent across cohorts, as well as generation-specific items that capture contemporary manifestations. Baby Boomer versions include items on work-related and family-structure contexts, reflecting these contexts. Generation Z versions include social media, climate anxiety, and digital identity items reflecting contemporary concerns. Generation Alpha versions include AI-related items that address dependency, authenticity, and the quality of the human-AI relationship.

Item Response Theory provides a statistical framework for creating these parallel forms while maintaining score comparability. Anchor items functioning equivalently across cohorts link forms, allowing placement on common scales. Generation-specific items capture unique experiences without forcing cross-generational comparison of non-equivalent content. This approach enables both within-generation assessment using entirely relevant content and cross-generation comparison through psychometrically sound linking procedures.

Within this IRT framework, candidate anchor items must first be rigorously screened for DIF using IRT-based procedures, particularly Rasch or 1-PL IRT models. These models estimate item parameters independently of specific normative populations, making them especially suited to cross-generational and cross-cultural applications where sample composition varies. Only items demonstrating DIF-free performance across cohorts qualify as valid anchors. These items then link all parallel forms to a common measurement scale. A calibrated item bank, constructed from validated anchor items and continuously monitored for emerging DIF as new cohorts are assessed, provides the most robust infrastructure for generation-spanning and culture-spanning psychological assessment. This unified psychometric framework connects linguistic drift directly to statistical non-invariance. As semantic meanings shift across cohorts, standard items inevitably exhibit DIF. Consequently, simple cross-cultural or cross-cohort adaptation of existing tools is insufficient. Instead, a calibrated item bank utilizing Rasch or 1-PL IRT models allows researchers to detect DIF-free anchor items. These anchors subsequently support Linear Logistic Test Model (LLTM) approaches that map cognitive processes, ensuring construct comparability while accommodating cohort-specific item development. This cross-cultural item bank should be treated as a living resource: updated as generational contexts evolve, expanded as new cohort-specific items are validated, and routinely re-evaluated for anchor stability.

### 5.2 Mandatory measurement invariance testing and longitudinal designs

All cross-generational research should adopt standardized invariance testing as a prerequisite before concluding that generational differences exist. The testing process proceeds hierarchically through increasingly stringent requirements. Configural invariance tests whether factor structure remains equivalent across groups. Metric invariance tests whether factor loadings are equal across groups. Scalar invariance tests whether item intercepts are equal across groups. Only when scalar invariance is achieved are valid comparisons of means across groups possible.

Confirmatory factor analysis provides the statistical framework for these tests. Researchers fit increasingly constrained models and compare model fit at each level. A significant decrease in fit indicates invariance failure at that level, suggesting that items function differently across groups. Chi-square difference tests, Comparative Fit Index (CFI) differences, and root mean square error of approximation (RMSEA) comparisons evaluate whether constraints significantly worsen fit.

When full scalar invariance fails, partial invariance models allow some items to vary while constraining others. This approach identifies which specific items exhibit DIF while enabling comparison on the invariant portion. Researchers should report which items require freeing and interpret the substantive meaning of non-invariance. Items showing DIF often reveal the most interesting generational differences, indicating where constructs manifest differently.

A complementary, particularly rigorous approach is the Linear Logistic Test Model (LLTM), which applies Rasch model principles to the theoretical structuring of test development. The LLTM enables researchers to model the specific cognitive operations hypothesized to govern item responses and to test whether those operations remain invariant across cohorts. Unlike standard DIF analysis, which confirms only that item difficulties are statistically comparable, the LLTM provides evidence that the cognitive processes underlying responses are themselves equivalent across generations. This provides strong evidence of construct comparability that CFA-based approaches alone cannot guarantee. Structural equation modeling offers an alternative path for cognitive validation of DIF-free items; however, SEM-based approaches may be unstable under small-sample conditions or in the face of the dynamic generational variation central to this manuscript (Ulitzsch et al., 2023). For researchers working within rapidly changing generational contexts, the LLTM therefore represents the more analytically stable option.

Cross-sectional designs cannot disentangle age, period, and cohort effects that confound generational comparisons. Longitudinal designs that track multiple cohorts over time enable the separation of these influences. Accelerated longitudinal designs measure multiple age cohorts over several years, creating overlapping age coverage. Cohort-sequential designs follow multiple cohorts longitudinally, directly observing how different birth cohorts traverse the same ages.

### 5.3 Implications for clinical practice, policy, and research

Clinicians using assessment tools validated on earlier cohorts with contemporary clients risk systematic misclassification. Test selection should prioritize instruments validated for the client’s generational cohort. When using older instruments, reports should acknowledge their limitations and interpret results cautiously. Apply the most recent cohort-specific norms rather than outdated published norms. Interpret scores using context-sensitive frameworks, considering generational contexts.

Diagnostic decision-making must recognize that Diagnostic and Statistical Manual of Mental Disorders, fifth edition (DSM-5) criteria may manifest differently across cohorts. Consider generation-specific presentations alongside traditional criteria. For Generation Z anxiety, evaluate climate anxiety and digital stressors. For Generation Alpha, consider AI-related dynamics in relationships and concerns about authenticity. Treatment planning requires adapting evidence-based interventions to generational contexts.

Policy implications extend to education, healthcare, and research systems. Educational testing requires regular test revisions and norm updates to account for AI-era learning. Mental health screening in schools needs updated cutoffs for current cohorts and generation-specific risk factors. Research funding agencies should require measurement invariance plans for any cross-cohort research and prioritize funding for instrument development and validation.

## 6 Discussion

### 6.1 Summary of key findings

The analyses presented here converge on the proposition that cross-generational psychological research suffers from fundamental measurement validity issues, with consequential implications for the existing literature. Each generation develops within unique technological, economic, and social contexts that alter how psychological constructs manifest, how questionnaire items should be interpreted, and what constitutes normative functioning. The Silent Generation, Baby Boomers, Generation X, Millennials, Generation Z, and Generation Alpha each represent a distinct psychological ecology that requires generation-specific assessment approaches.

Our analysis identified four core methodological problems: linguistic drift causes identical terms to mean different things across cohorts; context-dependent construct manifestations mean that supposedly universal phenomena actually vary by generation; outdated normative standards misclassify contemporary responses as pathological; and systematic violations of Differential Item Functioning theoretically invalidate direct cross-cohort score comparisons. These problems exist across all psychological domains, affecting depression, anxiety, social functioning, achievement motivation, self-esteem, attention, and every measured construct.

### 6.2 Linguistic drift as fundamental measurement threat

The semantic evolution of psychological terminology represents perhaps the most insidious threat to cross-generational validity because it operates invisibly. When the word “friend” appears identically on questionnaires administered to the Silent Generation and Generation Z, researchers assume semantic equivalence. However, this theoretical framework proposes that this term underwent a profound transformation in meaning (Perlman and Peplau, 1981; Parks and Floyd, 1996). What constitutes friendship, how friendships form and are maintained, and what they signify about social integration all changed fundamentally across these cohorts.

This problem extends beyond isolated terms to entire construct domains. The term “depression” evolved from a serious clinical descriptor to a casual colloquialism (American Psychiatric Association, 1952; Hidaka, 2012). Anxiety terminology shifted from specific fears to diffuse dread (Barlow, 2002; Horwitz and Wakefield, 2007). Definitions of success shifted from material markers to psychological fulfillment (Newman, 1993; Inglehart, 1977). Each semantic shift potentially compromises direct response comparisons, yet a substantial portion of cross-generational research proceeds without accounting for these changes.

The implications are profound. Studies reporting increased depression or anxiety rates in younger generations may reflect semantic drift rather than genuine increases in psychopathology. Without rigorous linguistic analysis and measurement invariance testing, these alternatives cannot be distinguished. The field may have inadvertently built a substantial portion of its cross-generational literature on measurement artifacts.

### 6.3 Context-dependent construct manifestations

Beyond linguistic drift, this conceptual analysis suggests that psychological constructs manifest differently across generations, challenging the assumption of construct universality underpinning psychological measurement. Loneliness demonstrates this clearly: the construct captured by 1970s scales measuring physical community integration has limited relevance for Generation Z, who experience “loneliness in crowds” amid hundreds of digital connections (Wellman et al., 2003; Rainie and Wellman, 2012; Chou and Edge, 2012; Harter, 2012).

Similarly, attention constructs developed for analog environments, which measure sustained single-task focus, mischaracterize Generation Z’s adapted cognitive strategies for high-stimulation digital contexts (Barkley, 1997; Ophir et al., 2009; Carrier et al., 2015). What appears to be attention deficit may reflect an evolved attentional distribution suited to contemporary demands. Depression constructs assuming future hopefulness indicates mental health fail to account for Generation Z’s rational assessment of climate catastrophe and economic decline (Beck et al., 1996; Wallace-Wells, 2019; Ojala, 2012).

These context-dependent manifestations violate the fundamental requirement of measurement theory that constructs remain stable across assessed groups. When Depression A (experiencing sadness despite favorable circumstances) differs from Depression B (experiencing despair about documented environmental collapse), comparing Depression Inventory scores across these groups lacks meaning. The field must either accept that constructs are historically situated rather than universal or develop measurement approaches that account for contextual variation.

### 6.4 Generation alpha: the tipping point

We hypothesize that Generation Alpha represents a qualitative departure that could render current measurement frameworks theoretically inadequate. While previous generational transitions involved gradual technological and social changes, Generation Alpha’s AI-integrated development from infancy creates fundamentally new psychological phenomena requiring conceptualization and measurement (Turkle, 2011; Livingstone and Blum-Ross, 2020; Palfrey and Gasser, 2008; Sparrow et al., 2011; Wechsler, 2008; Amabile, 1996; Bowlby, 1969; Clark and Chalmers, 1998). Their cognition may develop increasingly as a human-AI system rather than strictly individual capacity. Their social relationships include AI companions alongside human connections. Their creative and intellectual development begins with AI assistance.

Memory assessments may have reduced validity when external information storage is ubiquitous and adaptive (Sparrow et al., 2011). Social competence evaluations designed for face-to-face interaction miss digital-native strategies. Generation Alpha also necessitates the development of entirely new constructs. AI Relationship Quality, Cognitive Sovereignty, Synthetic Content Literacy, Digital Authenticity Comfort, Extended Intelligence Management, AI-Mediated Self-Concept, and Existential Displacement Anxiety are only initial conceptualizations of phenomena that require measurement.

The field must acknowledge that Generation Alpha’s psychological reality differs fundamentally from assessment frameworks developed in the 20th century, necessitating a comprehensive reconceptualization.

### 6.5 Implications for current research literature

The methodological problems identified in this conceptual analysis raise questions about a substantial body of published research on generational trends in mental health. Studies reporting that Generation Z experiences unprecedented anxiety and depression rates (Twenge et al., 2019a,b; Auxier and Anderson, 2021; Vuorre et al., 2021) may reflect measurement artifacts rather than genuine psychological differences. Without measurement invariance testing, semantic drift accounting, baseline recalibration, and context-sensitive interpretation, these conclusions remain unsubstantiated.

Systematic reviews and meta-analyses that synthesize cross-generational research inherit these validity problems from the primary studies (Twenge et al., 2010). Meta-analytic effect sizes comparing generations assume construct equivalence and measurement invariance, which are unlikely to hold. The field has potentially reached a premature consensus about generational mental health crises based on methodologically inadequate research.

Clinical implications follow directly. If clinicians believe Generation Z experiences elevated psychopathology based on invalid research, they may overdiagnose when symptoms actually reflect normative cohort functioning or adaptive responses to genuinely threatening environments (Holman et al., 2014; American Psychiatric Association, 2013). Policy interventions allocating substantial resources to youth mental health treatment may misattribute the problem when environmental change, measurement artifacts, or reporting bias better explain observed trends.

### 6.6 Strengths and limitations

This manuscript’s primary contribution lies in its broad theoretical analysis of measurement validity challenges across psychological domains and generational cohorts. We examined linguistic, psychometric, contextual, and interpretive dimensions rather than focusing narrowly on single instruments or constructs. The integration of measurement theory, generational context analysis, and available DIF evidence supports the central arguments advanced here.

However, several limitations warrant acknowledgment. As a Hypothesis and Theory contribution relying on narrative literature analysis, our literature selection reflects author judgment rather than a systematic search protocol. While we examined key studies demonstrating measurement non-invariance, a comprehensive systematic review of all DIF research across instruments would strengthen conclusions. Our analysis focuses primarily on North American and Western European generational contexts. Cross-cultural generational research introduces additional complexity that we did not fully address.

Empirical research directly testing our hypotheses remains limited. While existing DIF studies support our arguments (Twenge et al., 2010; Donnellan et al., 2009; Teresi and Fleishman, 2007; Glaesmer et al., 2008; Meade and Lautenschlager, 2004), comprehensive measurement invariance testing across all primary psychological instruments and generational cohorts has not yet been conducted. Our theoretical analysis identifies problems requiring empirical verification through large-scale psychometric studies.

Notably, this hypothesis and theory article does not address Generation Beta (projected birth years 2025–2039), the cohort currently being born. This omission reflects practical constraints rather than theoretical oversight. Generation Beta remains in its earliest infancy or is not yet born, precluding empirical assessment or developmental observation. However, this cohort will likely face measurement challenges even more pronounced than those identified for Generation Alpha. They will develop in environments where AI integration has progressed further, climate change impacts have intensified, and technological acceleration has continued. The measurement frameworks we propose for Generation Alpha will require adaptation and expansion for Generation Beta. Future research must remain vigilant to emergent psychological phenomena as this cohort develops, ensuring that assessment tools evolve alongside rapidly changing developmental contexts. The temporal lag between instrument development and cohort maturation creates persistent challenges. We urge the field to adopt proactive monitoring systems that track Generation Beta development from infancy, documenting novel psychological phenomena as they emerge, rather than retrospectively developing measurement frameworks.

### 6.7 Future research directions

The field requires a coordinated, systematic research program addressing identified measurement problems. First, priority should be given to comprehensive measurement invariance testing of all major psychological assessment instruments across generational cohorts. This work should employ rigorous multi-group confirmatory factor analysis testing configural, metric, and scalar invariance, with transparent reporting of DIF and partial invariance results (Cheung and Rensvold, 2002; Widaman and Reise, 1997).

Second, generation-specific instrument development programs should proceed systematically, beginning with qualitative research within each cohort to explore contemporary manifestations of the construct. This foundation enables the development of parallel forms with generation-appropriate content linked via IRT methods. Priority instruments include depression, anxiety, loneliness, self-esteem, achievement motivation, and attention measures, given their extensive use in cross-generational research.

Third, longitudinal research employing accelerated longitudinal and cohort-sequential designs must replace cross-sectional approaches to disentangle age, period, and cohort effects (Glenn, 2005). Only through following multiple cohorts across developmental periods can the field achieve valid conclusions about generational differences versus developmental changes.

Fourth, the development of assessments for Generation Alpha requires immediate attention, given their recent entry into educational and clinical systems. This includes both adapting existing constructs to AI-integrated contexts and developing entirely new measures for emergent phenomena like AI Relationship Quality and Cognitive Sovereignty. Interdisciplinary collaboration with computer science, education, and developmental psychology will prove essential.

Finally, the field needs updated psychometric reporting standards that require measurement invariance testing before any cross-generational comparisons. Journals should mandate invariance testing, transparent DIF reporting, and context-sensitive interpretation for all research comparing psychological constructs across cohorts separated by substantial temporal or technological change.

## 7 Conclusion

This theoretical analysis advances the argument that cross-generational psychological comparisons using standardized instruments with uniform interpretive frameworks may lack fundamental validity. Each generation develops within unique contexts that fundamentally alter how psychological constructs manifest and how assessment items function. A critical theoretical distinction emerges: core human psychological capacities remain fundamentally stable, but their historically situated manifestations, operationalizations, and appropriate measurement approaches vary by generation. Depression, anxiety, and motivation represent timeless aspects of psychology, yet how these constructs express themselves, what environmental factors trigger them, and how researchers can validly measure them differ profoundly across generations. Using assessment tools developed in the 1970s to evaluate children in 2025 digital environments exemplifies the methodological confusion plaguing the field. Contemporary five-year-old children code, use tablets, and process information through ubiquitous technology, representing fundamentally different developmental contexts than populations for which current instruments were validated. We argue these differences likely constitute massive confounding variables when researchers treat generation as a simple demographic covariate rather than a fundamental contextual variable. Where invariance has been tested, violations are consistently observed; semantic drift, baseline calibration shifts, and economic transformation each independently compound cross-generational measurement invalidity. Generation Alpha represents a paradigm case of measurement inadequacy, as its AI-integrated development constitutes a qualitative departure that may require a reconceptualization of intelligence, creativity, social competence, and autonomy, with entirely new psychological constructs potentially emerging. Addressing these challenges requires coordinated field-wide commitment to generation-appropriate instrument development, mandatory invariance testing, and context-sensitive interpretation. Without such commitment, cross-generational psychological research risks perpetuating findings of unverified validity with consequential implications for clinical and policy decisions.
